# Local control of polymicrobial infections via a dual antibiotic delivery system

**DOI:** 10.1186/s13018-018-0760-y

**Published:** 2018-03-15

**Authors:** David J. Tennent, Stefanie M. Shiels, Jessica A. Jennings, Warren O. Haggard, Joseph C. Wenke

**Affiliations:** 1 United States Army Institute of Surgical Research, 3855 Roger Brooke Drive, Fort Sam Houston, San Antonio, TX 78234 USA; 2 Department of Orthopaedics and Rehabilitation, San Antonio Military Medical Center, 3855 Roger Brooke Drive, Fort Sam Houston, San Antonio, TX 78234 USA; 3 Herff College of Engineering, 328D Engineering Technology Building, Memphis, TN 38152 USA

**Keywords:** Chitosan, Antimicrobial, Polymicrobial

## Abstract

**Background:**

Contaminated traumatic open orthopedic wounds are frequently complicated by polymicrobial contamination and infection. In high-risk wounds, the standard of care comprises debridement and irrigation combined with antibiotics which can be applied directly or combined with systemic antibiotics. Recently, bioabsorbable chitosan sponges have been shown to be an effective single-agent delivery device for local antibiotics with and without negative pressure wound therapy (NPWT). Severely contaminated orthopedic wounds, however, are often complicated by polymicrobial infections, necessitating multiple antibiotic agents. As such, the purpose of this study was to determine if a chitosan sponge would provide a suitable delivery vehicle for multiple antibiotics for the treatment of a polymicrobial infection in a large animal polytraumatic extremity wound model.

**Methods:**

A complex polytraumatic extremity wound was created in 11 adult male Boer goats. Each wound was contaminated with a bioluminescent strain of *S. aureus* (1 ml of 10^8^ colony forming units/ml) and of *P. aeruginosa* (1 ml of 10^8^ CFU/ml) which are genetically engineered to allow quantification with a photon-counting camera. Six hours following initial wound creation and contamination, wounds were debrided and irrigated with low-pressure normal saline. The animals were randomized into one of two treatments: wet-to-dry dressings alone or a commercially available chitosan sponge loaded with 1 g vancomycin and 1.2 g of tobramycin. Each animal was then recovered and reimaged 48 h later for total bacteria content; tissue samples were taken from the wound bed to determine relative bacterial colonization.

**Results:**

All animals in the chitosan sponge group saw significant reductions in overall bacterial load of *S. aureus* and *P. aeruginosa* (*p* = 0.001). The bioluminescence was also significantly reduced compared to the wet-to-dry dressing group (*p* = 0.0001). Furthermore, whereas the antibiotic sponge group displayed near complete eradication of bacteria, the wounds treated with the wet-to-dry dressings alone displayed a significant 2-log increase in total bacteria at 48 h *p* = 0.0001). *S. aureus* was the predominant species found in the wounds, comprising 95 and 99% of all bacteria found in the chitosan sponge and wet-to-dry, respectively.

**Conclusion:**

Dual antimicrobial therapy loaded in a chitosan sponge is an effective way to reduce polymicrobial infections traumatic extremity wound.

## Background

Traumatic, open orthopedic wounds are associated with high rates of patient-related morbidity and costs due to their mechanism of injury and prevalence of wound complications secondary to infection [1–5]. Specifically, high-energy lower extremity fractures have infection rates around 20% but have been reported as high as 52% [1–4]. The current standard of care for these injuries is debridement and irrigation combined with systemic antibiotics. Previous studies have shown that, amongst other factors, time to surgical debridement, time to definitive soft tissue coverage, and time to antibiotics correlate with infection rates following traumatic orthopedic trauma [6–9].

Many injuries are not amenable to the systemic delivery of antibiotics due to the severity of the soft tissue zone of injury and corresponding microvascular and macrovascular injuries. As such, a strong interest in local antibiotic delivery mechanisms by direct local application of antibiotic powders or via antibiotic carriers that allow a slower release of antibiotic over time is gaining in popularity [10–16]. Previous animal studies have shown that commercially available absorbable chitosan sponges are suitable carriers for single-agent antibiotic delivery and allow for reductions in bacteria with or without negative pressure wound therapy adjuncts when applied to a contaminated wound [10, 17]. These bioabsorbable chitosan sponges are capable of time-releasing antibiotics directly into a wound bed while also acting as a space filler [10, 17–20]. However, many traumatic orthopedic wound infections are polymicrobial and may require multiple antibiotics for adequate treatment. The purpose of this study was to determine whether chitosan sponges provide a suitable delivery mechanism for the local application of dual antibiotic therapy in the setting of a polymicrobial infection following a simulated traumatic extremity wound.

## Methods

This study was conducted in compliance with the Animal Welfare Act, the implementing Animal Welfare Regulations, and in accordance with the principles of the Guide for the Care of and Use of Laboratory Animals. All procedures were performed in a laboratory accredited by the Association and Accreditation of Laboratory Animal Care following a protocol approved by the Institutional Animal Care and Use Committee of the US Army Institute of Surgical Research.

A complex polytraumatic extremity wound was created in 11 adult male Boer goats (44 kg ± 2.7_SEM_, K Bar Livestock LLC, Sabinal, TX) to simulate a severe traumatic orthopedic related injury [10, 21]. On the day of surgery, animals received glycopyrrolate (0.01 mg/kg, SC) and carprofen (4.4 mg/kg, SC) approximately 1 h before surgery. Anesthesia was induced by a combination of ketamine hydrochloride (2.75 mg/kg) and midazolam (0.25 mg/kg), given intravenously. Anesthesia was maintained with 1–3% isoflurane via endotracheal intubation. A caudal epidural injection of morphine (0.1 mg/kg) was given at the initial surgery. A trapezoidal full thickness skin flap starting at the tibia tubercle and 8 cm in length, 5 cm wide proximally, and 3 cm distally was made in a single lower hind leg. To replicate a severe musculoskeletal injury, 13 g of anterior tibialis was excised to create a volumetric muscle loss injury; periosteum was removed leaving a 6-mm-wide strip; a 12-mm circumferential unicortical defect was created in the anterolateral tibia; a thermal burn on the bone, periosteum, muscle, fascia, and skin was made using electrocautery; and a 1 × 4-cm freeze injury was made using a liquid nitrogen cooled metal bar held directly on the surrounding musculature in two 30 s intervals.

Following creation of the severe orthopedic injury, each wound was contaminated with 10^8^ colony forming units (CFU) each of bioluminescent strains of *Staphylococcus aureus* (Xenogen 36; Caliper Life Science, Hopkinton, MA) and *Pseudomonas aeruginosa* (lux*)*, which are genetically engineered to allow quantification with a photon-counting camera as they release photons as a part of normal respiration. Bioluminescent bacteria emit light in proportion to their number, therefore allow noninvasive quantification using an external sensor. These bacteria were independently spread evenly over the entire wound bed using a cotton-tipped applicator. Each wound was then dressed with a sterile compressive, impermeable dressing. The animals were recovered in their cage until irrigation and debridement (I&D). Animals received once daily carprofen (4.4 mg/kg SC) for pain throughout the course of the study.

Baseline data was collected 6 h following initial wound creation and contamination. Each goat was again placed under general anesthesia in a light-free chamber that allows quantification of bacterial photon emission using a photon-counting camera (Charge Couple Device Imaging System Model C2400, Hamamatsu Photonics, Inc., Hamamatsu City, Japan). This camera allows for a quantitative and qualitatively determination of the amount and location of bacteria within a wound at the time of imaging. Briefly, a bright field photo was acquired of the area to establish wound borders. Then a photon image was acquired using the photon-counting camera which provides a visual representation and quantitative concentrations of photons within the area. The bright field and photon images were overlaid to create the region of interest and bioluminescence acquired using the Wasabi (Hamamatsu Photonics) interface software. After baseline bioluminescence was determined, each wound was irrigated with 9 l of low-pressure normal saline combined with mechanical soft tissue debridement that removed approximately 75% of the bacteria from the wound.

Each animal was randomly assigned to one of two treatment groups: the control group received a standard wet-to-dry dressings (WTD; *n* = 6) and the experimental group received a local treatment with a commercially available chitosan sponge (Sentrex Biosponge, Bionova Medical Inc., Germantown, TN) loaded with two clinically relevant antibiotics (CH-ABX; *n* = 5). The WTD dressing used sterile gauze and saline placed into the wound and covered with a sterile, impermeable dressing. The CH-ABX dressing was composed of a prepackaged 10 × 15-cm chitosan sponge (Sentrex Biosponge, Bionova Medical, Germantown, TN) capable of absorbing 0. 4 ml/cm^2^. The chitosan sponge was rehydrated with 60 ml of 0.9% normal saline mixed with 1 g vancomycin and 1.2 g tobramycin. The impregnated sponge was then placed directly into the contaminated wound and covered with an impermeable dressing. Each dressing was left in place until the end of the study.

All animals were anesthetized and euthanized 48 h after the initial time of contamination and bacteria again quantified using a photon-counting camera [10, 21]. Wound cultures were taken from the wounds systematically from the 12, 3, 6, and 9 o’clock positions within each wound. An additional culture was taken from an area identified as having the highest concentration of bioluminescence at the time of final imaging. Bacteria from these biopsies were then enumerated by plating serial dilutions of tissue homogenate supernatant onto three bacterial growth media: a non-selective 5% sheep blood agar, a CHROMagar that preferentially grows *S. aureus*, and a *Pseudomonas* Isolation agar that preferentially allows for *P. aeruginosa* growth [22]. The CFU were averaged across the wound and were normalized to tissue mass to determine the bioburden of the wound. Bioburden composition was determined as the ratio of *S. aureus* or *P. aeruginosa* to the total number of bacteria within the wound.

### Exclusion criteria

Animals were excluded from the study completely if they met one of the following criteria: dressing fell off before completion of the study, tibia or adjacent bones fractured due to surgical complications, animals were euthanized prematurely due to a failure to thrive, animals were found dead prior to completion of study, and baseline bacteria quantities were significant outliers based on Grubb’s test for outliers.

### Statistical methods

All data were analyzed using descriptive statistics, a *t* test, or a two-way ANOVA with Sidak’s multiple comparison test as appropriate. Significance was set at *p* < 0.05. A Grubb’s test was used to detect outliers. All data are presented as mean ± standard error of the mean.

## Results

**Fig. 1:**
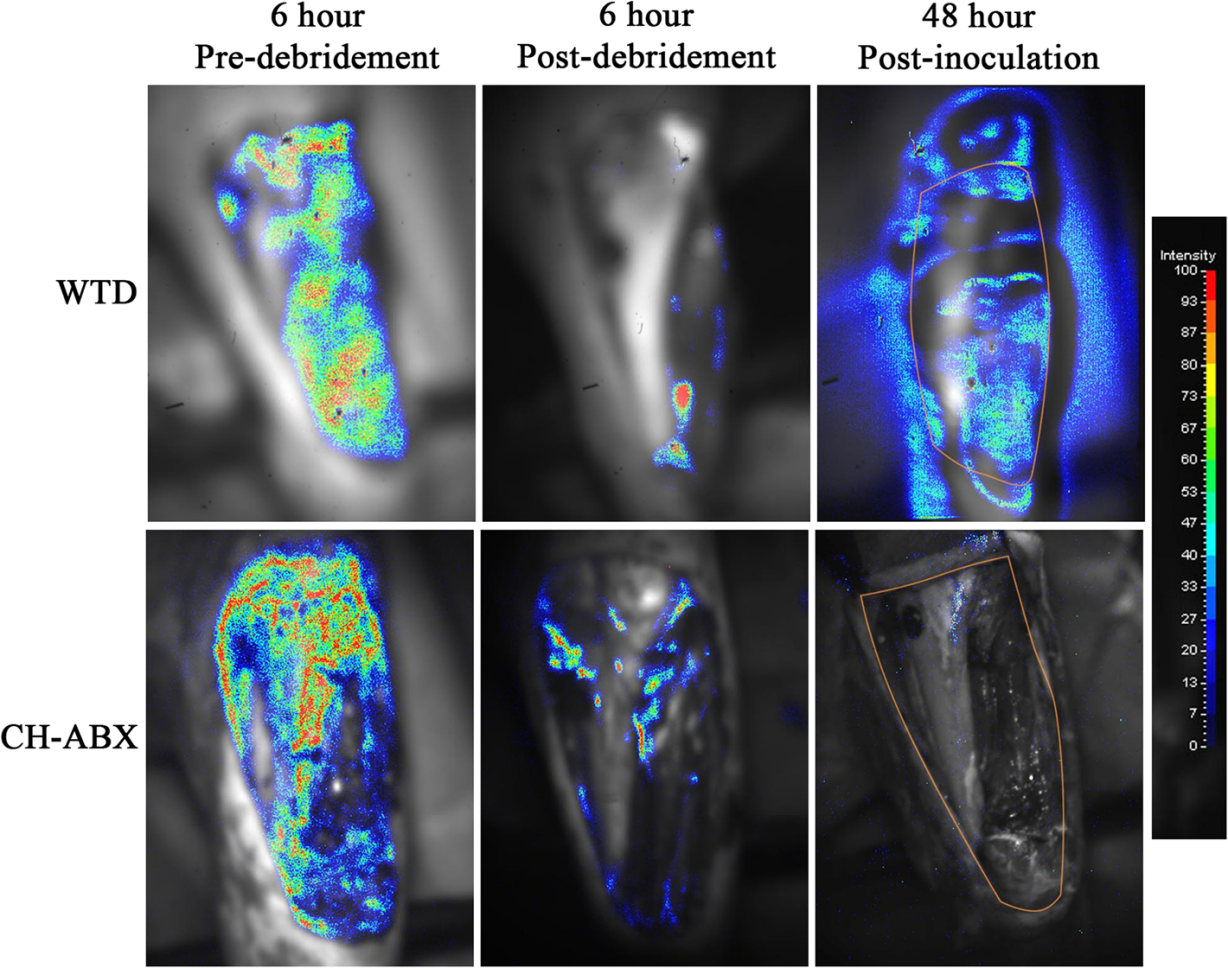
Representative black and white images with bioluminescent overlay. Higher intensity regions (red) correlate with increased bacteria load. Images from the left to right represent pre-debridement at 6 h from primary surgery, immediate post-debridement and irrigation with removal of approximately 75% of bacteria, and 48 h following primary surgery and inoculation. WTD wet-to-dry dressing, CH-ABX chitosan sponge with antibiotics

**Fig. 2:**
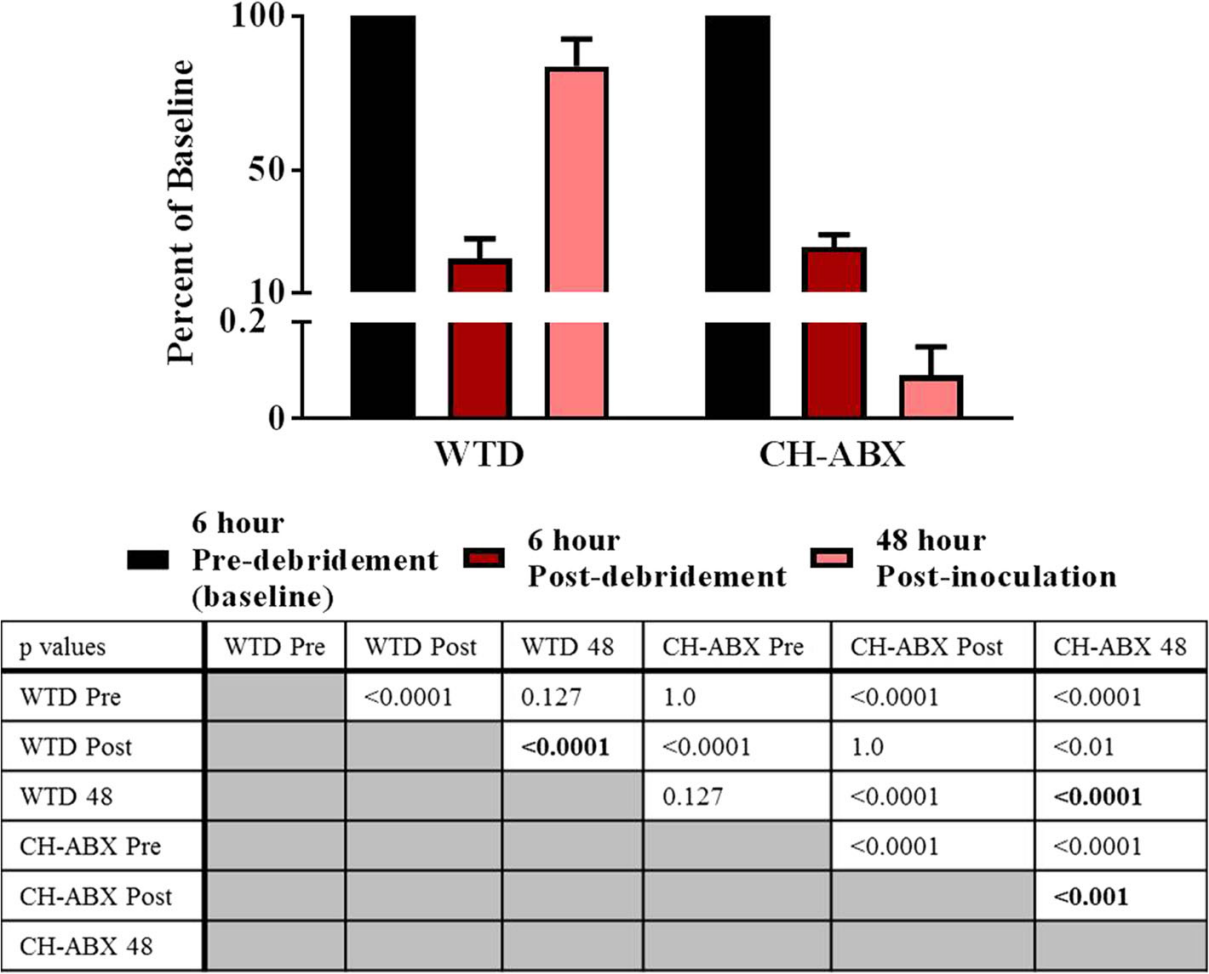
Percent bacteria remaining compared to initial imaging as found by bioluminescence. Baseline values are normalized to 100%, and each repeated measure is compared to its own baseline. A two-way ANOVA with a Sidek’s multiple comparison test was used. *P* values within the chart are bolded within the table and are of special interest

An overall decrease in bacteria was detected when the CH-ABX treatment was applied in lieu of the standard WTD dressing (Fig. 1). Within the control WTD dressing group, one animal died prior to completion of the study of undetermined causes, one animal was euthanized due to a fracture of the tibia shaft during wound creation, and an additional animal was found to be an outlier (Grubb’s test for outliers) and excluded completely from the study analysis as it displayed luminescence of 1000 fold of baseline. Using photoluminescence, it was determined that both groups had similar levels of bacterial contamination after the initial debridement, 6 h after contamination (controls 21.83 ± 4.89% baseline, CH-ABX 24.98 ± 3.99% baseline) (Fig. 2). All animals treated with CH-ABX survived and showed significant reduction in bacteria (0.09 ± 0.06% baseline, *p* < 0.001) where a near 100% bacterial reduction was observed (Fig. 2) between the post-debridement and the 48-h post-inoculation points. Compared to the WTD dressing control group, the CH-ABX significantly reduced the bacteria (*p* < 0.0001). At the time of final imaging, control animals showed a significant 2-log increase (83.60 ± 8.95%, *p* < 0.0001) in bacteria at 48 h (Fig. 2). Bacterial enumeration showed that 98.9 ± 1.1 and 94.7 ± 5.3% of bacteria cultured from the WTD and CH-ABX, respectively, were *S. aureus* (Table 1).

**Table 1:** Results of wound cultures on bacteria specific media

	Animals with detectable *S. aureus*	Percent of total enumerated	Animals with detectable *P. aeruginosa*	Percent of total enumerated
WTD	3/3	98.9*	3/3	1.1
CH-ABX	4/5	94.7*	1/5	5.3

*Represents significant differences at *p* < 0.0001 using *t* test comparing recovered bacteria percentage of *S. aureus* to *P. aeruginosa* within the same treatment type

## Discussion

Using an established contaminated traumatic orthopedic wound model, this study found that the application of a chitosan sponge containing vancomycin and tobramycin decreased bacterial burden in a polymicrobial wound. Furthermore, the antibiotic loaded chitosan sponges released the antibiotics at sufficient doses to achieve near complete eradication of bacteria 48 h following application. These treatment effects were not seen in the control group that had 2-log increase in bacterial counts when compared to post-debridement.

Prior studies have demonstrated that high-dose antibiotics are effective at treating and preventing infection in contaminated and high-risk orthopedic wounds [10–14, 16]. Specifically, a chitosan sponge has been shown to be an effective carrier of a single antibiotic against a single bacterial agent in previous studies using this model [10]. These studies have also shown that antibiotic levels above the MIC for *S. aureus* and *P. aeruginosa* are achievable when antibiotics are delivered via a chitosan sponge in both in vitro and animal studies [17, 19, 20]. This current study displayed similar results in a larger animal polymicrobial traumatic orthopedic wound model as bacteria were nearly entirely eradicated from the wounds treated with a vancomycin- and tobramycin-loaded chitosan sponge. This study also corroborates the use of a chitosan sponge for the use of polymicrobial infections and/or contaminated fracture care where a polymicrobial environment is expected. Furthermore, unlike antimicrobial loaded PMMA beads where the antimicrobial effect is limited to its immediate surroundings, the chitosan sponge is able to deliver a high dose of antibiotic over a large area within the complex wound. This may be particular of importance in those unstable patients who require repeated formal debridement and irrigation or in those patients who are not hemodynamically stable enough to undergo a thorough initial operative debridement. This delivery mechanism may also be useful in those cases where a negative pressure wound dressing is used as it has been shown to be more effective than PMMA beads or a negative pressure wound dressing alone [23].

This study has several limitations inherent to its preclinical animal model design. Most notably, this study only used a single treatment group with an untreated control. While this does impart a degree of potential bias in our results, this study was performed as a follow-up of previous work at our institution that used the same bacteria and model that has evaluated several other treatment modalities with reproducible success [10, 21, 23]. Repeating this study in successfully treated prior groups is not the best stewardship of laboratory animals and funding. The number of animals used in this study is small; however, the results are sufficiently dichotomous and statistically significant to the degree that continuing with additional animals was not required. This study employed a systematic sampling pattern in order to decrease any sampling bias that would occur with directed samples or random samples alone. While more stringent, this method does cause some degree of detection error and does not address specific areas of injury within the wound. As such, imaging directed samples were obtained at areas of highest bioluminescence in order to decrease our detection error and increase the probability that we would successfully obtain qualitative data. Lastly, this study only evaluated the treatment effects at 48 h. Prior work has shown that the majority of the antibiotic is released within the first 72 h [20]. As such, the remaining sponge may prove to be a nidus for infection if not removed. However, this is not unlike PMMA where the carrier necessitates removal in subsequent surgeries and does not allow for definitive management [24, 25].

## Conclusion

This study suggests that the chitosan sponge is an effective dual antibiotic carrier that can be used effectively to combat a persistent infection in a polymicrobial-infected traumatic orthopedic wound. The treatment effect seen in this study is consistent with previous studies evaluating single-agent treatment and may prove to be a more effective carrier mechanism than antibiotic depots such as bone cement. While this study does provide promising results, further studies evaluating this carrier in comparison to other topically applied antimicrobials may be warranted.

## Abbreviations

CFU: Colony-forming units
CH-ABX: Chitosan sponge with antibiotics
CHROMagar: Chromographic agar
NPWT: Negative pressure wound therapy
*P. aeruginosa*: *Pseudomonas aeruginosa*
PMMA: Polymethylmethacrylate
*S. aureus*: *Staphylococcus aureus*
WTD: Wet-to-dry
